# Genetic Diversity of Circulating Foot and Mouth Disease Virus in Uganda Cross-Sectional Study During 2014–2017

**DOI:** 10.3389/fvets.2020.00162

**Published:** 2020-03-25

**Authors:** Lauro Velazquez-Salinas, Frank Norbert Mwiine, Zaheer Ahmed, Sylvester Ochwo, Anna Munsey, Julius J. Lutwama, Andres M. Perez, Kimberly VanderWaal, Elizabeth Rieder

**Affiliations:** ^1^Foreign Animal Disease Research Unit, USDA/ARS Plum Island Animal Disease Center, Greenport, NY, United States; ^2^College of Veterinary Medicine, University of Minnesota, Minnesota, MN, United States; ^3^College of Veterinary Medicine, Animal Resources and Biosecurity (COVAB), Makerere University, Kampala, Uganda; ^4^Department of Emerging and Re-emerging Diseases, Uganda Virus Research Institute, Entebbe, Uganda

**Keywords:** FMDV, VP1, Uganda, Cattle, Sequences

## Introduction

Foot and mouth disease (FMD) is one of the most economically devastating animal diseases, threatening the livestock industry around the world (1). FMD is caused by foot and mouth disease virus (FMDV), an RNA virus in the *Picornaviridae* viral family, genus *Aphthovirus*, from which seven different serotypes have been described (A, O, C, Asia 1, SAT 1, SAT 2, and SAT 3) (2). The existence of multiple topotypes and the lack of cross protection between serotypes are just some of the factors limiting the control and eradication of FMDV (3). Thus, it is imperative to continuously characterize FDMV genetic diversity in affected countries.

In Uganda, factors like uncontrolled animal movments, the existence of wildlife reservors, and poor vaccine performance have created conditions for FMDV to maintain endemicity since it was first reported there in 1953 (4–6). In terms of genetic diversity, recent reports demonstrate the presence of at least five out of the seven serotypes (A, O, SAT 1, SAT 2, and SAT 3) and multiple topotypes, affecting livestock across the country (4, 7–10). Historically, FMDV O has been one of the most prevalent serotypes in Uganda, the most recently report indicates the circulation of at least five different lineages (11).

In this context, the implementation of quarantines and vaccination programs have failed to control FMD in this country (12). Reports indicate that FMD clinical cases increased in Uganda during the 2000's relative to the 1990's (13). A recently risk analysis study showed the complexity involving the epidemiology of FMD in Uganda, being the proximity with international borders one of the most important factors associated with the circulation of FMDV in this country (14). Based on the sanitary conditions in east Africa, officially the export and import trade activities of livestock in Uganda is limited (1.5% all export values), but should be taken into account as a potential factor to favor the circulation of FMDV in the region, being Burundi, Democratic Republic of Congo, Kenya, Rwanda, Southern Sudan and Tanzania the major export markets (http://www.fao.org/3/a-at589e.pdf). The rapid evolution of FMDV in Uganda might be explained by a combination of evolutionary mechanisms characteristic of RNA viruses (recombination, positive, and negative selection, and random drift constraints), which all shape the quasispecies dynamics of endemic populations, thereby increasing the ability of this virus to rapidly adapt to different conditions in nature (15, 16). In this context, the continuous genetic characterization of circulating FMDV variants could support the development of more effective control strategies in this country (13).

Herein, we are reporting the availability of a valuable collection of a VP1 and P1 (complete capsid coding) protein coding region sequences in the GenBank database, representing the genetic diversity of FMDV from 29 districts representing different geographical regions in Uganda between 2014 and 2017 (Supplementary File 1).

The VP1 protein coding region is the genetic marker typically used to perform phylogenetic analyses and to group FMDV into specific genotypes, also referred as topotypes (17). The VP1 protein contains relevant antibody neutralizing sites and T and B-cell epitopes which have been the subject of multiple studies aimed at understanding the evolution of FMDV in response to immunological pressures (18–22)

## Methods

Esophageal-pharyngeal (“Probang”) sampling was part of a cross sectional study conducted in cattle herds in Uganda between 2014 and 2017 during a multidisciplinary research project supported by the Cooperative Biological Engagement Program of the U.S. Department of Defense Threat Reduction Agency, Defense Threat Reduction Agency. The research was conducted by experts from Plum Island Animal Disease Center (PIADC), University of Minnesota in the United States, University of Makerere, and the Virus Research Institute in Uganda.

After collection, probang samples were snap-frozen, and stored at −70°C at University of Makerere, until samples were sent to PIADC for testing. Sequencing work was conducted at PIADC in the United States. All viral sequences were obtained from viral isolations on cell monolayers of LFPKαVβ6 (one passage) (23). Isolates were from oropharyngeal fluid samples (probang samples) collected from naturally infected FMD cattle herds in Uganda between 2014 and 2017. (For more details about the location of each isolate see Supplementary File 1).

Viral RNA was isolated from cell culture supernatants using the RNeasy MiniKit (QIAGEN) and sequencing work was performed by the Sanger method following a protocol previously described, which includes the use of universal FMDV primers (24). Final consensus VP1 coding region sequences were obtained using Sequencher v4.8 (Gene codes, Ann Arbor, MI, USA). Based on the nucleotide homology, different sequences were classified into specific serotypes using the Blastin algoritm (25). Based on the nucleotide variability, for some of the viral isolations, the entire P1 coding region was obtained using a methodology previously described (26).

The viral sequence collection reported here is currently being analyzed in combination with sequences previously reported in East Africa in order to establish the phylogenetic relationships of recent viral lineages in this region. The aim of our work is to support the Ugandan authorities for the development of a risk-based approach to mitigate the impact of FMD in this country. Interestingly, for more than 25 years, Ugandan authorities have used a trivalent FMD vaccine containing serotypes O, SAT 1, and SAT 2, which is manufactured in Kenya (KEVEVAPI) (10). Information on the quality and potency of the vaccine is not available. Additionally, the vaccine is manufactured with fairly historic viral strains (GenBank access: *O* = K77/78; HM756588, SAT1 = T155/71; HQ267519, and SAT2 = K52/84; HM623685). The serotype O strain included in the vaccine is characterized as topotype EA-1, however recent reports have demonstrated inefficacy of the vaccine against FMDV serotype O, topotype East Africa two (EA-2), one of the most prevalent genetic lineages in Uganda (13, 27). In this context, our collection of viral sequences might support the selection of potential vaccine candidate strains to reformulate the current trivalent vaccine, and ultimately improve FMD control strategies in Uganda.

Furthermore, since very little is known about the evolutionary dynamics of different serotypes circulating in Uganda, this sequence collection is currently being used to identify specific sites in the capsid protein evolving under positive selection using a codon-based phylogenetic framework (28).

These results will help to choose appropriate viral lineages to support further work by next generation sequencing, which will increase our understanding about the contribution of different viral proteins in the evolution of different viral lineages in Uganda. Also, these extensive collection of viral sequences will represent an important reference for future phylogenetic analyses conducted in Uganda.

**Figure 1:**
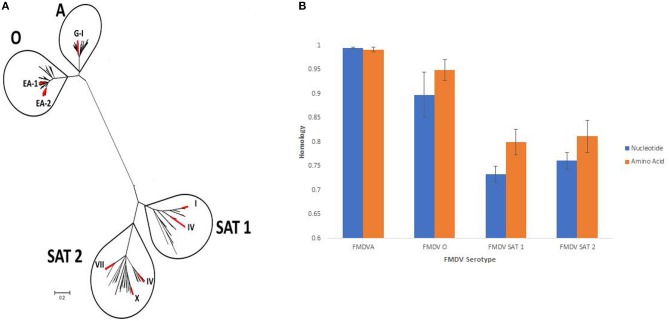
FMDV sequencing dataset from Uganda (2014–2017). **(A)** phylogenetic analysis conducted by maximum likelihood method, showing the genetic diversity of the FMDV sequencing dataset reported in this article. Multiple reference sequences from each serotype previously described by Knowles et al. (2) were included for this analysis. Branches in red represents specific topotypes associated with the sequences reported in this database. **(B)** Homology from each serotype at nucleotide and amino acid levels was deduced by pairwise distance analysis. In case of serotypes 0, SAT 1, and SAT 2 pairwise distance was calculated between different topotypes, thus explaining the disparate amino acid homology displayed between these serotypes. Analysis were conducted on the software MEGA 10.0.5.

Collectively, the purpose of this report is to announce the availability of this sequence dataset, which represents the genetic variability of FMDV in Uganda during 2014–2017, in public databases. The entire VP1 sequence datset collection from this project comprises a total of 258 sequences including serotypes A (*n* = 4) (topotype G-I), O (*n* = 148) (topotypes EA-1 and EA-2), SAT 1 (*n* = 70) (topotypes I and IV), and SAT 2 (*n* = 36) (topotypes IV, VII, and X). Information about the genetic diversity and homology at nucleotide and amino acid levels among the sequences within each serotipe contained in this data set is shown in Figures 1A,B, respectively. However, part of the collection (*n* = 117) was already used for initial phylogenetic analysis, and these sequences were reported elsewhere (11). To avoid possible duplications, here we are reporting the remaining sequences, comprised of 141 previously unpublished VP1 sequences representing serotypes O (*n* = 102) and SAT 1 (*n* = 50), as well as a total of 36 P1 sequences including serotypes O (*n* = 30) and SAT 2 (*n* = 6).

Genbank accession numbers and corresponding sequences are available in Supplementary File 1.

## Data Availability Statement

The datasets generated for this study can be found in the the Accession Genbank; number information for the sequence dataset collection is detailed in the Supplementary Material.

## Ethics Statement

The animal study was reviewed and approved by the Ethics Commitee of the College of Veterinary Medicine, Animal Resources and Biosecurity (COVAB), Makerere University, Kampala, Uganda. Written informed consent was obtained from the owners for the participation of their animals in this study.

## Author Contributions

ER, FM, JL, KV, and AP conceived the study. ER, FM, and JL obtained funding. LV-S and ZA performed virus sequencing/genomic analysis. AM and KV perform data interpretation. FM and JL performed sampling activities. LV-S and ER wrote the manuscript. All authors read and approved the manuscript content.

### Conflict of Interest

The authors declare that the research was conducted in the absence of any commercial or financial relationships that could be construed as a potential conflict of interest.

## Supplementary Material

Supplementary File 1 Accession Genbank number information form the entire VP1 and P1 sequence dataset collection representing the FMDV genetic diversity in Uganda between 2014 and 2017. Rows colored in green represent unreported sequences, while colored in blue represent sequences used for phylogenetic analysis reported in Mwiine et al. (11). Codes for vaccination status are represented by *N* = non-vaccinated, *Y* = vaccinated, and *U* = unknown. It is important to consider that majority of the sequences come from viral isolations conducted on cattle vaccinated most likely after being exposed to FMDV. To visualize potential FMDV serotype or topotype affinity, information was stratified by breed.
