# Supplementary figures and images for: Genetic Diversity of Circulating Foot and Mouth Disease Virus in Uganda Cross-Sectional Study During 2014–2017
Source: Front Vet Sci. 2020 Mar 25;7:162. doi: 10.3389/fvets.2020.00162 (PMC7109301; doi:10.3389/fvets.2020.00162)

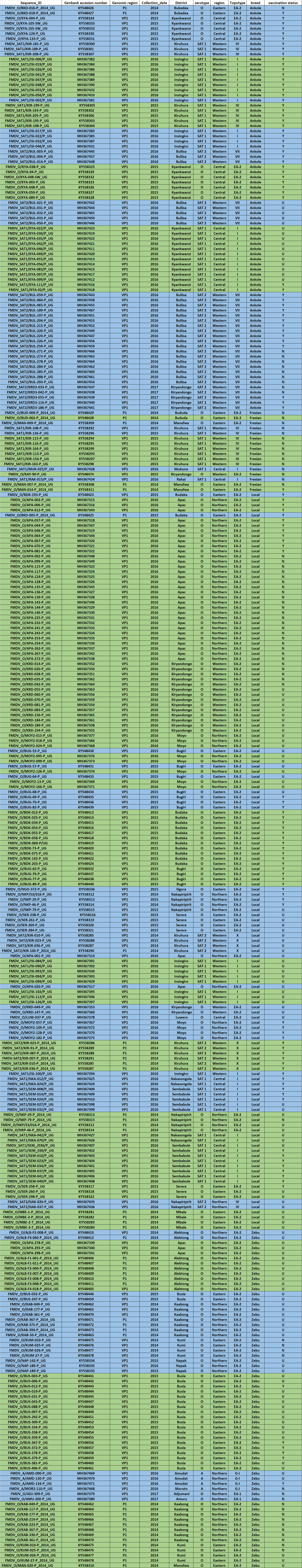

Supplement: Supplementary File 1 — Accession Genbank number information form the entire VP1 and P1 sequence dataset collection representing the FMDV genetic diversity in Uganda between 2014 and 2017. Rows colored in green represent unreported sequences, while colored in blue represent sequences used for phylogenetic analysis reported in Mwiine et al. (11). Codes for vaccination status are represented by N = non-vaccinated, Y = vaccinated, and U = unknown. It is important to consider that majority of the sequences come from viral isolations conducted on cattle vaccinated most likely after being exposed to FMDV. To visualize potential FMDV serotype or topotype affinity, information was stratified by breed. [file Image_1.TIF]
